# Corticotropin-releasing factor (CRF) in brain aging: from mitochondrial dysfunction to inflammaging

**DOI:** 10.3389/fnagi.2026.1880569

**Published:** 2026-07-31

**Authors:** Mengting Yan, Linrui Hua, Jufu Jiang, Jingyi Wu, Ziyan Zeng, Yun He, Yuncai Chen

**Affiliations:** Department of Anatomy, School of Medicine, Yangtze University, Jingzhou, China

**Keywords:** aging, cellular senescence, chronic inflammation, corticotropin-releasing factor (CRF), mitochondrial dysfunction, stress

## Abstract

Aging is a complex biological process. The corticotropin-releasing factor (CRF) signaling pathway has gained increasing attention for its potential role in regulating aging, acting as a key bridge connecting neuroendocrine stress mechanisms with both central and peripheral aging processes. As a core neuropeptide of the stress response, CRF primarily mediates downstream effects through its type 1 receptor (CRFR1), while its type 2 receptor (CRFR2) may play a modulatory, often opposing, role. Evidence from preclinical models indicates that excessive CRF signaling is associated with mitochondrial dynamics imbalance and biogenesis defects, leading to overproduction of reactive oxygen species (ROS) and mitochondrial DNA damage. These damaged mitochondria can subsequently release damage-associated molecular patterns (DAMPs), which activate innate immune pathways and may trigger a chronic low-grade inflammatory state, ultimately contributing to cellular senescence. Elucidating the cascading mechanisms by which CRF signaling could drive aging—from mitochondrial dysfunction to chronic inflammation—is essential for understanding the molecular basis of stress-accelerated aging. This review aims to systematically integrate research advances at the intersection of the CRF signaling pathway and aging hallmarks, focusing on a proposed “CRF-mitochondrial dysfunction-inflammation-aging” axis. We explore this regulatory network and its clinical translational potential to provide a theoretical foundation for unraveling the molecular mechanisms of stress-driven aging and identifying novel intervention targets.

## 1 Introduction

Aging is a complex biological process characterized by genomic instability, telomere attrition, epigenetic alterations, loss of proteostasis, impaired macroautophagy, dysregulated nutrient sensing, mitochondrial dysfunction, cellular senescence, stem cell exhaustion, altered intercellular communication, chronic inflammation, and dysbiosis (López-Otín et al., 2023). These hallmarks are interdependent and together constitute a networked driving force of the aging process (Nunkoo et al., 2024). Within the central nervous system (CNS), striking region-specific vulnerabilities to these processes have been documented: the hippocampus exhibits reduced neurogenesis and synaptic plasticity, the prefrontal cortex shows executive function decline, and the amygdala displays abnormal emotional processing (Mattson et al., 2008; Verkhratsky et al., 2012). Notably, these brain regions also represent core nodes of the stress-response neural circuitry, implying a profound mechanistic link between chronic stress exposure and accelerated brain aging.

Among these hallmarks, mitochondrial dysfunction, chronic inflammation, and cellular senescence constitute an interconnected triad that is centrally positioned at the intersection of neuroendocrine stress and brain aging. Mitochondrial dysfunction serves as a proximal target of CRF signaling, while the senescence-associated secretory phenotype drives chronic inflammation, and both senescence and inflammation in turn exacerbate mitochondrial damage, thereby forming a self-amplifying loop. Other hallmarks, such as telomere attrition or proteostasis loss, are less directly coupled to acute stress pathways in the context of neuroendocrine signaling (López-Otín et al., 2023; McEwen, 2017). Thus, focusing on this triad enables a cohesive mechanistic narrative that traces from an upstream trigger, CRF, to its downstream consequences. Unlike previous reviews that have addressed CRF in stress and neurodegeneration largely in isolation (Bangasser et al., 2017; Vandael and Gounko, 2019), the present review integrates these three pillars into a sequential framework linking CRF signaling to mitochondrial dysfunction, inflammation, and senescence, offering a testable model that distinguishes our approach from prior work.

**FIGURE 1:**
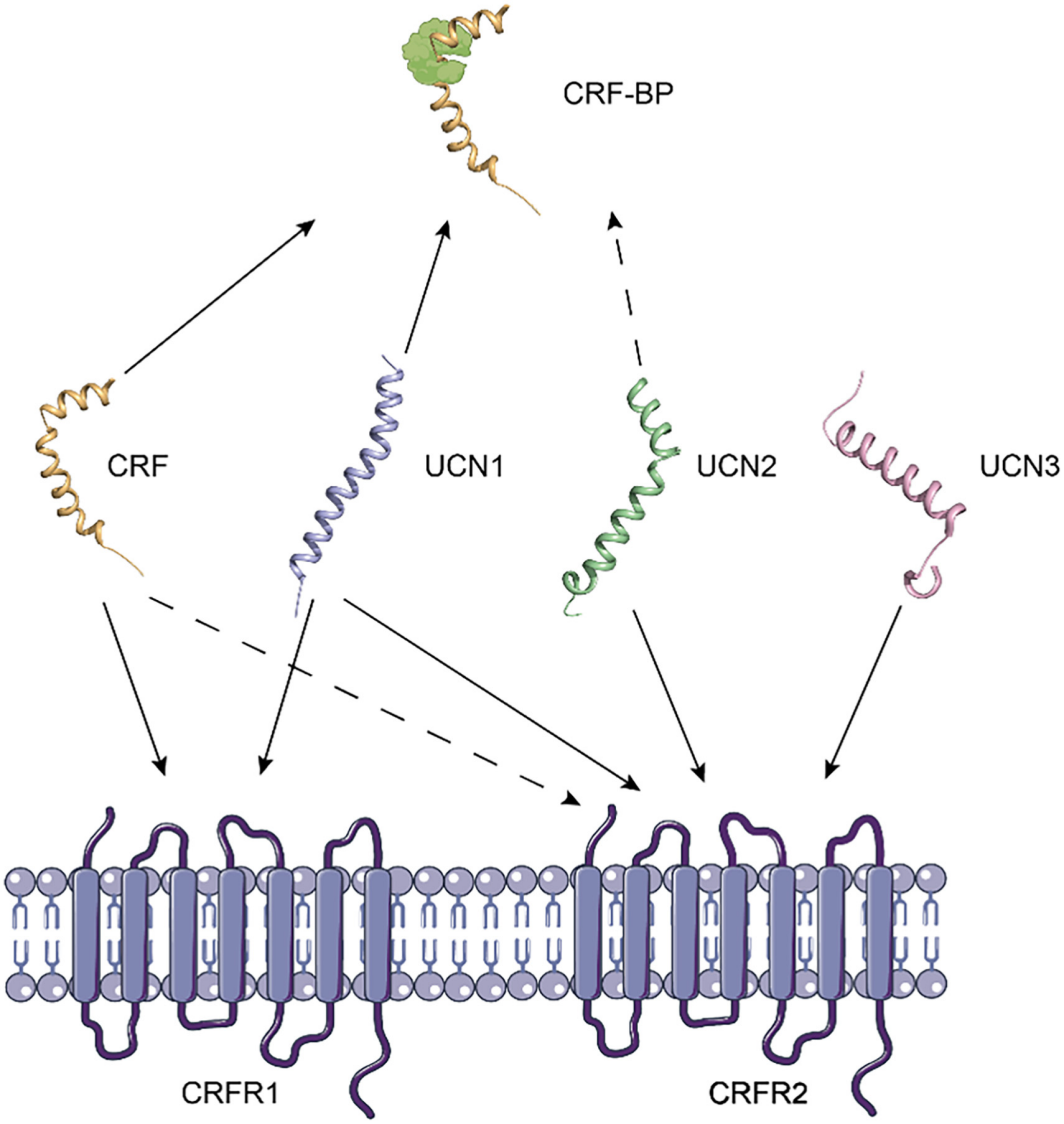
This diagram illustrates the relationship between the CRF-binding protein (CRF-BP) and the CRF peptide family (CRF, UCN1, UCN2, UCN3). Arrows indicate the affinity between peptide ligands and their receptors (solid arrows) or between peptide ligands and CRF-BP (dashed arrows). Different arrow line patterns denote varying degrees of affinity, with dashed arrows representing lower-affinity binding compared to solid arrows. CRF, corticotropin-releasing factor; CRFR1, CRF1 receptor; CRFR2, CRF2 receptor; UCN1, urocortin 1; UCN2, urocortin 2; UCN3, urocortin 3.

Corticotropin-releasing factor (CRF) is the principal initiator of the hypothalamic-pituitary-adrenal (HPA) axis and the core regulatory molecule of the neuroendocrine stress response (Vale et al., 1981; Smith and Vale, 2006; Kageyama and Suda, 2009). CRF is mainly synthesized in the paraventricular nucleus (PVN) of the hypothalamus, from which it stimulates adrenocorticotropic hormone (ACTH) release by activating pituitary corticotrophs, ultimately driving glucocorticoid (GC) secretion from the adrenal cortex (Mavridis et al., 2025; Kageyama et al., 2021). The CRF family comprises four ligands, including CRF, urocortin 1, urocortin 2, and urocortin 3. These ligands exert their biological effects by activating two G protein-coupled receptors, CRF receptor type 1 (CRFR1) and type 2 (CRFR2) (Figure 1; Friedman and Irwin, 1995; Reyes et al., 2001; Fudge et al., 2025). However, beyond this classical neuroendocrine cascade, CRFR1 and CRFR2 are widely expressed in the hippocampus, prefrontal cortex, amygdala, and locus coeruleus, where they regulate neuronal excitability, synaptic plasticity, and glial reactivity in a region-specific manner (Deussing and Chen, 2018; Chen, 2016; Dedic et al., 2018; Domin and Şmiałowska, 2024). This distributed brain CRF system can operate independently of peripheral HPA axis activation, thereby enabling local paracrine and autocrine stress signaling within neural circuits critical for cognitive and emotional regulation (Deussing and Chen, 2018). Importantly, the CRF system is not confined to central neuroendocrine regulation. Both ligands and receptors are widely distributed in peripheral tissues, including the heart, blood vessels, gastrointestinal tract, and skeletal muscle, where they influence systemic stress responses through immune and metabolic modulation (Bagosi et al., 2023; Stenmark and Graham, 2018).

Chronic stress has emerged as a major risk factor for accelerated brain aging and neurodegenerative diseases through sustained activation of the CRF-HPA axis (Vandael and Gounko, 2019; Kline and Mega, 2020; Ross et al., 2018). Clinical evidence, particularly from neuroimaging studies, indicates that long-term stress exposure is closely associated with hippocampal atrophy, prefrontal cortical thinning, and cognitive decline in the elderly (Lupien et al., 2009; Gold et al., 2015). At the molecular level, chronic stress elevates circulating glucocorticoids (GCs), which exert neurotoxic effects via glucocorticoid receptor (GR)-mediated genomic and non-genomic pathways, thereby impairing mitochondrial biogenesis, promoting neuroinflammation, and inducing cellular senescence (Gandy et al., 2023). However, emerging evidence suggests that CRF may also influence neuronal mitochondrial function and inflammatory tone through local CRFR1 signaling in neurons and glial cells, potentially operating in parallel with, or independently of, GC-mediated mechanisms (Bangasser et al., 2017; Reyna et al., 2023). This is a nuanced and actively debated area of research, but it raises the intriguing possibility that CRF acts as a more direct upstream modulator of multiple brain aging markers than previously appreciated. Nevertheless, the integrative regulatory role of CRF and the relative contribution of GC-dependent versus GC-independent pathways remain incompletely understood, and it is recognized that the effects attributed to CRF often occur alongside elevated glucocorticoids, which are themselves potent drivers of these aging hallmarks.

**FIGURE 2:**
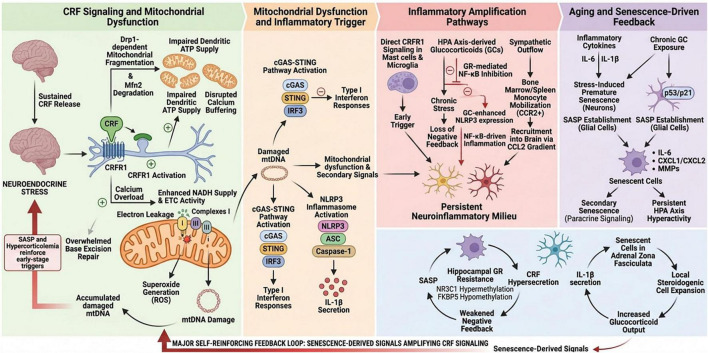
This diagram illustrates the proposed mechanistic framework integrating CRF signaling with mitochondrial dysfunction, neuroinflammation, and cellular senescence in stress-accelerated brain aging. Chronic stress activates the HPA axis, driving CRF release and initiating a self-amplifying loop that integrates mitochondrial dysfunction, neuroinflammation, and cellular senescence. CRF-CRFR1 signaling disrupts mitochondrial dynamics, leading to calcium overload, ROS generation, and mtDNA damage. Damaged mtDNA activates the cGAS-STING pathway, which primes the NLRP3 inflammasome and triggers type I interferon responses. Inflammation is amplified through three pathways: direct CRFR1 activation of mast cells and microglia; HPA axis-derived glucocorticoids that shift from anti-inflammatory to pro-inflammatory via GR resistance; and sympathetic-mediated CCR2^+^ monocyte recruitment into the brain. Sustained inflammation drives neuronal stress-induced premature senescence (p53/p21) and glial SASP (IL-6, CXCL1/CXCL2, MMPs). SASP factors and hypercortisolemia reinforce HPA axis hyperactivity through hippocampal GR resistance and epigenetic reprogramming (NR3C1 hypermethylation, FKBP5 hypomethylation), as well as through adrenal senescent cell-derived IL-1β, which stimulates CRF hypersecretion, closing the feedback loop.

Beyond the HPA axis, CRF signaling also directly impacts mitochondrial function in peripheral tissues, leading to excessive ROS production, oxidative stress, and activation of innate immune pathways, thereby establishing a detrimental “stress–mitochondrial damage–inflammation–aging” cycle. In this review, we focus on three primary aging hallmarks: mitochondrial dysfunction, chronic inflammation, and cellular senescence. We systematically dissect the molecular mechanisms by which CRF signaling may influence these processes, then integrate these findings into a unified framework that we term the “CRF–mitochondria–inflammation–senescence axis” (Figure 2). Finally, we discuss diagnostic and therapeutic strategies targeting this axis and outline future research directions to bridge the gap between mechanistic insights and clinical translation in stress-related aging and age-associated diseases.

## 2 CRF-associated mitochondrial dysfunction in the aging brain

Mitochondrial dysfunction is a hallmark of brain aging, characterized by mitochondrial DNA (mtDNA) mutation accumulation, impaired oxidative phosphorylation, excessive ROS production, and disrupted mitochondrial dynamics (Bratic and Larsson, 2013; Xu et al., 2025). In the aging brain, these defects are particularly pronounced in stress-vulnerable regions such as the hippocampus and prefrontal cortex, where mitochondrial bioenergetic failure contributes to synaptic loss and cognitive decline (Steffan et al., 2025). Emerging evidence suggests that CRF, traditionally viewed as the HPA axis initiator, may directly impair mitochondrial homeostasis in neural cells via CRFR1-mediated signaling, supporting a conceptual framework in which neuroendocrine signals intersect with mitochondrial aging pathways (Zhang and Rissman, 2017; Battaglia et al., 2020).

### 2.1 CRFR1-cAMP-PKA signaling disrupts mitochondrial dynamics and biogenesis

Upon binding to CRFR1, CRF activates the Gs protein, which triggers adenylyl cyclase activity, resulting in elevated intracellular cAMP levels and subsequent activation of protein kinase A (PKA) (Fernandez-Marcos and Auwerx, 2011; Grammatopoulos, 2012; Deussing and Chen, 2018). PKA-mediated phosphorylation of CREB normally up-regulates PGC-1α to promote mitochondrial biogenesis (Abu Shelbayeh et al., 2023). However, under chronic CRF exposure, this signaling cascade may be redirected toward mitochondrial fission. In hippocampal neurons, sustained CRFR1 activation leads to Drp1 phosphorylation at Ser616, a modification mediated by stress-activated kinases including CDK5 and ERK1/2, which drives mitochondrial fragmentation (Battaglia et al., 2020; Zhou et al., 2017). This transition from fusion to fission disrupts the integrity of the mitochondrial network required by dendrites, which is critical for supplying ATP and buffering calcium ions, thus making synapses vulnerable to excitotoxicity (Mattson et al., 2008).

In parallel, CRF may further contribute to the division process via the non-receptor tyrosine kinase c-Abl. c-Abl phosphorylates Drp1 at tyrosine residues Y266, Y368, and Y449, thereby enhancing its GTPase activity and promoting its translocation to the outer mitochondrial membrane (Zhou et al., 2017). Notably, mitochondrial dynamics are inherently malleable, and stress-induced fission states can be normalized upon restoration of homeostatic conditions (Twig et al., 2008), providing a potential therapeutic window for early intervention.

Corticotropin-releasing factor (CRF) signaling may also inhibit mitochondrial fusion through two potential mechanisms. First, Mfn2 can be degraded by stress-activated E3 ubiquitin ligases such as Parkin and Huwe1; however, direct evidence specifically linking CRF to this pathway is currently limited (Tanaka et al., 2010; Leboucher et al., 2012); Second, stress-activated OMA1 cleaves OPA1 to produce short OPA1 (S-OPA1), which loses its fusion ability (Anand et al., 2014). The resulting fragmentation could disrupt mitochondria-associated membranes (MAMs), hypothetically affecting calcium transfer from the endoplasmic reticulum to mitochondria and activating the unfolded protein response (UPR) (Urbani et al., 2020; Rizzuto et al., 2012). MAM dysfunction in the aging brain impairs lipid transfer and cardiolipin synthesis, leading to cristae structural disorganization and oxidative phosphorylation coupling defects, which have been closely linked to synaptic dysfunction and neuronal vulnerability in neurodegenerative diseases (Area-Gomez and Schon, 2017; Area-Gomez et al., 2018).

### 2.2 CRF-associated calcium overload and ROS generation

Corticotropin-releasing factor (CRF) activates ryanodine receptor (RyR)/inositol 1,4,5-trisphosphate (IP3) receptors and voltage-gated calcium channels (VGCCs) in a CRFR1-dependent manner, leading to mitochondrial calcium overload in certain neuronal populations (Riegel and Williams, 2008; Dautzenberg et al., 2004; Deussing and Chen, 2018). This overload enhances NADH supply and electron transport chain (ETC) activity, resulting in increased electron leakage and superoxide generation at complexes I and III (Angelova and Abramov, 2018; Zhao et al., 2019; Muntean et al., 2016). While these findings are derived from specific neuronal populations, they highlight a potential mechanism that may be relevant in other stress-vulnerable brain regions. In aging neurons, the calcium-ROS cycle forms a self-amplifying loop; ROS oxidize RyR and SERCA, further disrupting calcium homeostasis (Tadic et al., 2014). Meanwhile, ROS-damaged mtDNA accumulates due to overwhelmed base excision repair (BER) capacity (Stevnsner et al., 2002; Muftuoglu et al., 2014).

MtDNA damage, marked by 8-OHdG formation, is particularly detrimental given the high metabolic demands of brain tissue and the postmitotic nature of neurons (Lin and Beal, 2006; Wen et al., 2025). Compounding this, chronic stress may impair mitophagy—the selective autophagic clearance of damaged mitochondria—which would further promote the accumulation of defective mitochondria and the release of their oxidized mtDNA into the cytosol (Sliter et al., 2018). Critically, mitochondrial permeability transition pore (mPTP) opening or mitochondrial membrane disruption releases mtDNA to the cytoplasm, thereby activating STING-dependent type I interferon responses and NLRP3 inflammasome activation (Bradshaw et al., 2022; Wei et al., 2024). This mtDNA-driven neuroinflammation may constitute a molecular bridge linking CRF-associated mitochondrial dysfunction to the chronic inflammatory state that characterizes the aging brain.

### 2.3 Cell-type specificity: neurons and astrocytes

The effects of stress on mitochondrial dynamics show clear cell-type specificity in the CNS. In hippocampal neurons, chronic CRF exposure has been associated with Drp1-dependent mitochondrial fragmentation (Battaglia et al., 2020). Although neurons may temporarily maintain bioenergetic homeostasis, their mitochondrial tolerance declines with age, leaving aged neurons vulnerable to bioenergetic failure (Wen et al., 2025; Lin and Beal, 2006; Bratic and Larsson, 2013). In astrocytes, stress-induced mitochondrial dysfunction may impair the lactate shuttle mechanism that supports neuronal energy metabolism (Pellerin and Magistretti, 1994; Magistretti and Allaman, 2015). Senescent astrocytes exhibit reduced mitochondrial network connectivity and altered glutamate homeostasis, which may exacerbate neuronal vulnerability (Verkhratsky et al., 2012; Mattson et al., 2008). The resulting neurovascular unit dysfunction further compromises the integrity of the blood-brain barrier (BBB) under chronic stress (Sweeney et al., 2018; Zlokovic, 2011).

## 3 CRF-associated neuroinflammation and inflammaging

Chronic inflammation, one of the hallmarks of brain aging, is characterized by persistent microgliosis, astrocyte reactivity, and elevated proinflammatory cytokines, including IL-1β, IL-6, and TNF-α (Baechle et al., 2023; Franceschi et al., 2018). In the aging brain, this neuroinflammatory state, known as “inflammaging,” is exacerbated by chronic stress and dysregulated HPA-axis signaling (Ferrucci and Fabbri, 2018). CRF, as the main initiator of the HPA axis, may promote inflammation indirectly through GC resistance, while also potentially acting directly on CNS immune cells via CRFR1, thereby contributing to the persistent neuroinflammatory environment (Herman et al., 2016; Domin and Şmiałowska, 2024; Nunez et al., 2025; Bellavance and Rivest, 2014).

### 3.1 CRF promotes chronic inflammation through the HPA axis and GC resistance

As a key activator of the HPA axis, CRF is continuously released during chronic stress, leading to sustained HPA axis activation and increased GC secretion (Herman et al., 2016). Under normal conditions, GC exerts a potent anti-inflammatory effect through GR, inhibiting the production of proinflammatory cytokines such as IL-1β, IL-6, and TNF-α, while regulating immune cell function (Bellavance and Rivest, 2014). However, chronic stress leads to GC resistance, reduces the sensitivity of immune cells to GC, and impairs the anti-inflammatory response, ultimately promoting a proinflammatory state (Cohen et al., 2012). Notably, under certain conditions—particularly chronic stress—GCs can also exhibit proinflammatory properties, enhancing the expression and function of the NLRP3 inflammasome and promoting IL-1β secretion. This dual feature is context-dependent and especially evident under chronic stress conditions (Frank et al., 2010; Coutinho and Chapman, 2011).

Glucocorticoid (GC) resistance is characterized by downregulation of GR expression, altered GR function, and diminished effects on downstream signaling pathways (Walsh et al., 2021). Specific mechanisms include disruption of the GR-HDAC2 pathway, overexpression of TXNIP, and increased mitochondrial membrane permeability, which contributes to mtDNA release (Zhou et al., 2010; Nakahira et al., 2011). Under chronic stress, microglia and astrocytes in the brain exhibit dysfunction of GR signaling, making it difficult for GC to effectively inhibit proinflammatory transcription factors such as NF-κB, thereby perpetuating the inflammatory response in brain regions critical for stress integration, including the hippocampus, prefrontal cortex, and amygdala (Wohleb et al., 2011; Frank et al., 2012). In addition, excess cortisol enhances mineralocorticoid receptor (MR) binding, and activation of MR promotes the upregulation of IL-6 and TNF-α in immune cells such as macrophages, which may further aggravate the proinflammatory state (Chapman et al., 2013).

Stress-activated GCs can promote neuroinflammation through the GR-NF-κB-NLRP3 signaling pathway (Frank et al., 2012; Oakley and Cidlowski, 2013). Chronic restraint stress increases cortisol levels in the hippocampus, downregulates GR expression, and triggers nuclear translocation of NF-κB (Iwata et al., 2016; Munhoz et al., 2006). As a transcriptional activator of NLRP3, NF-κB mediates inflammasome activation, while ROS and mitochondrial DNA act as secondary activation signals (Bauernfeind et al., 2009; Zhou et al., 2011). This process promotes the assembly of the NLRP3 inflammasome and the maturation and release of IL-1β and IL-18, which continuously maintains a chronic inflammatory state in the CNS (Martinon et al., 2006). This signaling cascade is critical for neuropsychiatric disorders associated with abnormal HPA axis regulation and is increasingly recognized as a driver of age-related cognitive decline (Ding et al., 2025; Heneka et al., 2013).

### 3.2 Non-HPA axis mechanisms: direct and sympathetic pathways

In addition to mechanisms mediated by the HPA axis, CRF may drive neuroinflammation through two distinct potentially pathways independent of GC signaling. The first pathway involves direct activation of CNS-resident immune cells, whereas the second relies on sympathetic nervous system-mediated recruitment of peripheral immune cells.

CRF directly activates mast cells and microglia through CRFR1-mediated signaling (Theoharides et al., 2016). In mast cells that functionally express CRFR1, CRF acts in synergy with degranulation stimuli to enhance histamine and tryptase release while inducing endogenous CRF synthesis, thus establishing an autocrine amplification loop that disrupts the BBB (Ayyadurai et al., 2017). In microglia, CRF has been reported to upregulate galectin-3 and disrupt autophagy through a CRFR1-dependent mechanism, potentially promoting a proinflammatory phenotype characterized by IL-1β and TNF-α release; however, further studies are needed to establish the directness and mechanistic details of this pathway. At the same time, CRF stimulates sympathetic outflow and increases circulating catecholamine levels. Sympathetic activation enhances the release of inflammatory monocytes from the bone marrow through downregulation of CXCL12 expression and promotes the release of monocytes from the spleen through β-adrenergic receptors (Heidt et al., 2014; Hu and Zeng, 2018). While both bone marrow- and spleen-derived monocytes can be mobilized, the relative contribution of each compartment to stress-induced neuroinflammation in the brain has not been systematically compared. It is hypothesized that they both contribute to the persistent neuroinflammatory environment. Thus, whereas the direct pathway elicits local inflammation within the CNS, the sympathetic pathway provides additional inflammatory effectors from the periphery.

In contrast to the predominantly proinflammatory role of CRFR1, the role of CRFR2 in neuroinflammation is more complex and often appears to be opposing. CRFR2 has been shown to mediate anti-inflammatory effects and promote stress recovery in some contexts (Dermitzaki et al., 2018). However, its specific contribution to the aging brain remains poorly understood. The balance between CRFR1 and CRFR2 signaling, and their cell-type-specific expression, likely determines the net inflammatory outcome. This underscores the need for future studies to dissect the distinct functions of these two receptor subtypes in the context of aging. Despite the growing interest in these non-HPA axis mechanisms, whether CRFR1 and CRFR2 mediate distinct microglial phenotypes in the aging brain has not been clearly demonstrated.

### 3.3 Integration: context-dependent inflammatory outcomes

The HPA axis, direct neuroimmune pathways, and sympathetic pathways described above do not operate in isolation, but rather intersect with each other to establish a self-sustained inflammatory environment in the aging brain. However, the overall inflammatory output of CRF signaling depends largely on the specific context. For example, acute CRF release induces a transient anti-inflammatory effect via HPA-axis-derived GCs, whereas chronic activation leads to GR tolerance and the opposite pro-inflammatory GC effect. Similarly, CRFR1 activation generally promotes proinflammatory responses, whereas CRFR2 signaling may play a regulatory or anti-inflammatory role under certain conditions (Dermitzaki et al., 2018). This bidirectional regulation may explain why the effects of global CRF blockers vary according to disease stage, tissue context, and duration of stress exposure, with some reducing inflammation while others increasing it (Kehne and Cain, 2010).

Several unanswered questions limit the translational potential of therapies targeting the CRF-neuroinflammation axis. There is ongoing controversy regarding whether central versus peripheral sources of CRF exert distinct effects on brain inflammaging (Kovács et al., 2019). In addition, whether CRFR1 inhibitors can selectively suppress neuroinflammation without affecting systemic stress-adaptive mechanisms requires careful evaluation. Future studies using cell-type-specific CRFR knockout models and longitudinal immune-cell tracking may help to dissect the interplay of these pathways and identify age-related therapeutic windows (Ising et al., 2007).

## 4 CRF-associated cellular senescence and SASP amplification

Cellular senescence is a significant indicator of aging. Its characteristics include cell cycle arrest, resistance to apoptosis, and development of the senescence-associated secretory phenotype (SASP) (Wang et al., 2024). As a central regulatory element of the neuroendocrine stress response, CRF may promote aging-related processes through both direct receptor-mediated signaling pathways and indirect glucocorticoid elevation, thus representing a key node linking stress to brain aging.

### 4.1 Neuronal senescence and impaired neurogenesis

When considering senescence in the brain, it is important to clarify that the concept of cellular senescence encompasses distinct states, particularly in post-mitotic neurons. While proliferating cells undergo classical cell cycle arrest, post-mitotic neurons, which cannot divide, do not become senescent in the same way. Instead, they can acquire a “senescence-like” phenotype in response to severe stress, characterized by the secretion of proinflammatory factors and the expression of senescence-associated markers such as p21 and p16 (Jurk et al., 2012).

Neurons may undergo stress-induced premature senescence (SIPS) through activation of the classical p53-p21 and p16-Rb pathways (de Keizer et al., 2010). Chronic activation of the CRF-HPA axis increases GC levels, which in turn triggers a DNA damage response and mitochondrial dysfunction, potentially contributing to senescence-like changes in post-mitotic neurons (Fu et al., 2024; de Keizer et al., 2010). There is an environment-dependent link between GCs and aging. Although acute glucocorticoid exposure can trigger cell-cycle arrest in some proliferating cell types, chronic exposure may selectively inhibit some components of the SASP without reversing the aging state, as has been observed in fibroblast models (Fu et al., 2024). At the molecular level, GCs inhibit PGC-1α expression through GR-mediated genomic effects, thereby hindering mitochondrial biogenesis and promoting ROS activates the p53/p21 signaling pathway and initiates a DNA damage response, which in turn promotes cellular senescence (Qin et al., 2024; Fu et al., 2024).

CRF-associated GC elevation further inhibits adult hippocampal neurogenesis and reduces neuronal differentiation and survival by down-regulating BDNF levels and affecting GR nuclear translocation in neural progenitor cells (Tsimpolis et al., 2024; Suri and Vaidya, 2013; Mahar et al., 2014). The resulting decrease in neurogenesis impairs the brain’s ability to replace damaged neurons and accelerates functional decline in stress-susceptible regions (Cruceanu et al., 2022).

Epigenetic mechanisms further link chronic stress to neuronal aging. Hypermethylation of the hippocampal NR3C1 promoter and hypomethylation of FKBP5 intron 7, both associated with early-life adversity, reduce GR expression and cortisol sensitivity, respectively, resulting in a dual impairment of negative feedback that perpetuates HPA-axis hyperactivity (McGowan et al., 2009; Klengel et al., 2013). This epigenetic reprogramming may lead to accelerated brain aging and increased susceptibility to neurodegenerative diseases (Zannas et al., 2019; Alexander et al., 2020).

### 4.2 CRF amplifies senescence via SASP and systemic feedback loops

Hyperactivity of the HPA axis caused by chronic stress accelerates the aging of glial cells, transforming astrocytes and microglia into persistent sources of SASP and thus amplifying neuroinflammation (Melo Dos Santos et al., 2024; Pessoa et al., 2026). In astrocytes, stress-induced mitochondrial dysfunction may disrupt lactate transport between astrocytes and neurons, limiting metabolic support to neurons (Pellerin and Magistretti, 1994; Wang et al., 2026; Preeti et al., 2022). Recent evidence suggests that aging astrocytes undergo a metabolic shift from aerobic glycolysis to oxidative phosphorylation, with upregulated LDHB expression and reduced lactate supply to neurons, exacerbating neuronal energy deficits (Acevedo et al., 2023). Aging astrocytes progressively show reduced mitochondrial network connectivity as well as altered glutamate homeostasis, which may exacerbate neuronal vulnerability (Olajide, 2025; Pessoa et al., 2026).

Senescent microglia exhibit reduced phagocytic capacity and increased release of SASP components, including IL-6, CXCL1/CXCL2, and MMP3/MMP12, thereby amplifying neuroinflammation in the neural network (Rawji et al., 2016). SASP factors create a self-sustaining inflammatory environment by inducing secondary senescence of neighboring cells through paracrine signaling (Admasu et al., 2021; Teo et al., 2019).

Systemically, this aging-driven inflammation forms a feedback loop that maintains HPA axis hyperactivity through two mechanisms: centrally, chronic GC exposure promotes GR tolerance in the hippocampus, weakening negative feedback inhibition and leading to excessive CRF secretion (Froger et al., 2004; Laryea et al., 2015); peripherally, senescent cells accumulate in the zona fasciculata of the adrenal gland and secrete IL-1β as part of SASP, acting locally to promote excessive cortisol secretion (Okudaira et al., 2024). This feedback loop suggests that stress can not only initiate senescence-like changes in the brain but also perpetuate them.

### 4.3 Evidence for systemic CRF-aging effects beyond the CNS

The effects of CRF signaling on aging-related processes are not limited to the traditional HPA axis target organs. In skin, local CRF and CRFR1 expression levels increase with age, while CRF-BP expression decreases, resulting in long-term exposure of dermal fibroblasts to altered CRF signaling (Slominski et al., 2013). CRF has been shown to directly trigger human hair follicle cells to enter a senescence-like state, characterized by increased SA-β-gal activity and down-regulation of growth phase gene expression (Lee et al., 2024), which may provide a potential mechanism for stress-related alopecia. These findings suggest that CRF-associated aging processes operate in peripheral tissues through both systemic endocrine pathways and local paracrine/autocrine mechanisms, providing evidence for a systemic CRF-aging axis.

## 5 Mechanisms of CRF-mitochondria-inflammation-aging axis integration and intervention strategies

This section integrates the preceding mechanistic evidence into a unified framework, synthesizes the diagnostic potential of CRF signaling, and evaluates therapeutic strategies targeting this axis.

### 5.1 The integration mechanism of the CRF-mitochondrial-inflammation-aging axis

The mechanisms detailed in Sections 2–4 converge into a self-reinforcing axis linking stress to brain aging. In this framework, CRF acts as a core molecular hub: it disrupts mitochondrial homeostasis, triggers innate immune activation, and establishes feedback loops that propagate senescence. Sustained CRFR1 activation induces mitochondrial fragmentation and calcium overload, leading to ROS generation and mtDNA damage. Damaged mtDNA released into the cytosol activates cGAS–STING and NLRP3 inflammasome pathways, bridging mitochondrial dysfunction to neuroinflammation.

This inflammatory response is amplified through three converging pathways: direct CRFR1 signaling in glial and immune cells, HPA-axis-derived glucocorticoids that shift from anti-inflammatory to pro-inflammatory under chronic stress, and sympathetic-mediated recruitment of CCR2^+^ monocytes. The resulting neuroinflammatory microenvironment, combined with chronic GC exposure, promotes senescence-like changes in neurons and SASP establishment in glial cells. SASP factors propagate secondary senescence through paracrine signaling, while senescent cells in the adrenal gland and GC-induced GR resistance in the hippocampus reinforce HPA axis hyperactivity. Epigenetic reprogramming (NR3C1 hypermethylation, FKBP5 hypomethylation) further perpetuates this cycle. Thus, the axis operates as a positive feedback loop in which CRF-associated mitochondrial damage and inflammation promote senescence, and senescence-derived signals in turn reinforce excessive CRF secretion.

### 5.2 The potential value of CRF signaling as a diagnostic marker for age-related diseases

The multi-system nature of the CRF-mitochondria-inflammation-senescence axis indicates that CRF and its downstream signaling molecules may serve as integrated biomarkers for age-related diseases. Unlike traditional single markers, such as telomere length or p16INK4a, which capture only one dimension of cellular aging (Géranton, 2019), CRF levels reflect the dynamic interplay between psychological stress, HPA axis activity, and systemic inflammation.

In the field of neurodegenerative diseases, CSF biomarkers play a key role in the diagnosis and monitoring of Alzheimer’s and Parkinson’s diseases (Pingle et al., 2023; Cao et al., 2025). The CRF system is closely related to the pathology of Alzheimer’s disease. CRF promotes the production of Aβ by regulating the activity of γ-secretase (Park et al., 2015), and CRFR1 antagonist can alleviate oxidative stress and neuronal damage in Alzheimer’s disease transgenic mouse models, thereby improving the pathological manifestations of Aβ and cognitive impairment (Zhang and Rissman, 2017). By measuring CRF levels and CRFR1 activity in peripheral blood, clinical assessment is expected to assess chronic stress and aging. By assessing the expression of CRFR1 and the phosphorylation level of NF-κB in relevant tissues, the degree of local inflammatory aging and tissue damage can be measured.

However, the current clinical translation faces many limitations. Peripheral CRF levels can be affected by acute stress and drugs, resulting in significant fluctuations. Meanwhile, disease-specific expression patterns differ between tissues and pathological stages. Future research should focus on multi-center longitudinal studies, the establishment of standardized detection protocols, the exploration of combined application with other aging markers, and the development of multi-dimensional diagnostic models combining CRF signal with mitochondrial function indicators and SASP spectrum.

### 5.3 Aging intervention and treatment strategies targeting the CRF pathway

By targeting the CRF–CRFR1 signaling axis and its downstream pathways, it is possible to intervene concurrently in mitochondrial dysfunction, chronic inflammation, and cellular senescence. This section focuses primarily on CRF-targeted interventions, while briefly considering complementary approaches.

#### 5.3.1 Drug intervention targeting the CRF system

CRFR1 antagonists demonstrate potential for anti-stress and anti-aging effects in animal models; however, significant challenges hinder their clinical translation. Antalarmin, an early non-peptide CRFR1 antagonist, effectively blocked stress-induced elevations in ACTH and exhibited antidepressant-like effects in rats (Jutkiewicz et al., 2005). Nonetheless, both Antalarmin and CP-154,526 failed to show antidepressant activity in the forced swim test, possibly due to behavioral inhibitory effects or limited sensitivity of this test to such compounds. Pharmacokinetic analyses indicate that early CRFR1 antagonists possess excessive lipophilicity, contributing to increased toxicity and reduced bioavailability (Spierling and Zorrilla, 2017).

Pexacerfont (BMS-562086) demonstrated a lack of efficacy in trials for generalized anxiety disorder and failed to alleviate cravings or anxiety in studies of alcohol dependence (Lee et al., 2023; Oatu et al., 2025). Although pexacerfont effectively binds to central CRFR1 receptors, with cerebrospinal fluid concentration predicted to achieve nearly 90% occupancy, this did not translate into clinical efficacy. Its rapid dissociation kinetics are believed to contribute to this lack of effectiveness (Spierling and Zorrilla, 2017). In contrast, verucerfont (NBI-77860/GSK561679) differs from pexacerfont primarily in receptor retention duration; verucerfont exhibits a longer receptor retention time, which diminishes ACTH and cortisol responses while attenuating amygdala fMRI activation (Schwandt et al., 2016). Nevertheless, verucerfont has not demonstrated clinical efficacy in trials for depression, PTSD, or alcohol dependence. Importantly, among women with PTSD, the effect of verucerfont was not significant in the general population (Koob and Zorrilla, 2012), but rather in a subgroup of women characterized by a history of childhood abuse and GG homozygosity for CRFR1 rs110402 (Dunlop et al., 2017).

For CRFR1 antagonists, poor physical and chemical properties of early candidates, insufficient specificity of preclinical screening models, and positive results confined to specific conditions are key limitations. Neuroadaptive changes occurring after long-term CRFR1 activation may also reduce the need for continued antagonism (Spierling and Zorrilla, 2017). Future advancements should focus on precision medicine strategies, particularly biomarker-guided stratification based on CRFR1 gene polymorphisms (rs110402) and childhood trauma history. Exploring indication repurposing is also crucial, including potential applications in congenital adrenal hyperplasia (21-hydroxylase deficiency), where verucerfont has demonstrated effectiveness in reducing ACTH and 17α-hydroxyprogesterone levels. Notably, crinecerfont, a CRFR1 antagonist, received FDA approval for classical CAH treatment in December 2024, marking the first new drug in this area in 70 years (U.S. Food and Drug Administration, 2024, 2025). Combining CRFR1 antagonists with SSRIs, SNRIs, or cognitive-behavioral therapy may boost efficacy, with careful monitoring of pharmacokinetic interactions. Developing allosteric modulators rather than competitive antagonists represents a promising direction (Giatro et al., 2025), and tissue-selective modulators may help avoid peripheral side effects (Tanaka et al., 2017). Sustained-release formulations should be developed to ensure stable receptor occupancy and improve drug delivery precision.

#### 5.3.2 Mitochondrial-targeted antioxidants

Mitochondrial-targeted antioxidants offer a complementary approach to alleviate CRF-associated mitochondrial damage. SS-31 (elamipretide) binds to cardiolipin, stabilizes mitochondrial cristae structure, alleviates oxidative stress, and enhances ATP production (Szeto et al., 2017). Clinical trials have shown improvement in exercise capacity and cardiac function in Barth syndrome patients after extended treatment (Reid Thompson et al., 2021; Patai et al., 2025). However, clinical translation faces challenges including short-term efficacy limitations, subcutaneous injection requirements with associated site reactions, and heterogeneous tissue responses (Ehlers et al., 2025). Future directions include optimizing treatment cycles, developing oral formulations, and combining with NAD^+^ precursors or senolytics.

#### 5.3.3 Senescent cell clearance strategy

Targeted elimination of senescent cells has emerged as a strategy to disrupt the detrimental cycle of CRF and aging (Saliev and Singh, 2025). The combination of dasatinib and quercetin (D+Q), has been validated for safety and efficacy in multiple clinical trials, including diabetic nephropathy, idiopathic pulmonary fibrosis, and Alzheimer’s disease (Hickson et al., 2019; Justice et al., 2019). A phase II trial showed that intermittent senolytic treatment significantly increased bone formation markers in postmenopausal women with high aging load (Farr et al., 2024). However, challenges remain, including dose-dependent side effects and heterogeneity in aging mechanisms across populations (Barthet and Lowe, 2025). Next-generation strategies, including immune senolysis (e.g., CAR-T targeting uPAR) and tissue-specific PROTACs, are emerging (Sun et al., 2019). Caution is necessary when applying senolytics in cardiovascular diseases due to potential adverse effects (Yang et al., 2025).

#### 5.3.4 Lifestyle intervention

Lifestyle regulation offers a non-pharmacological approach to modulate the CRF-aging axis. Regular exercise promotes mitochondrial biogenesis through PGC-1α activation (Abrego-Guandique et al., 2025). Intermittent fasting and calorie restriction enhance mitophagy via PINK1/Parkin and BNIP3 pathways. Optimizing sleep can restore normal cortisol secretion patterns (Tripathi et al., 2021; O’Byrne et al., 2021). The combined application of these interventions alongside pharmacological treatments may represent a promising avenue for future anti-aging strategies.

## 6 Discussion

This review systematically integrates the association between CRF and three fundamental aging hallmarks: mitochondrial dysfunction, chronic inflammation, and cellular senescence. We propose an integrated regulatory network termed the “CRF-mitochondria-inflammation-senescence” axis, with CRF as a primary upstream node. Through a comprehensive analysis of current literature, we suggest that CRF serves not only as a traditional stress hormone but also as a pivotal molecular nexus linking psychological stress to biological aging mechanisms. Importantly, we present this framework as a conceptual model to guide future hypothesis-driven research, rather than as an established consensus.

### 6.1 Limitations and unresolved questions

significant portion of the evidence cited in this review is derived from animal models or *in vitro* experiments, with limited direct validation of the CRF-aging axis in humans. Throughout this review, we have endeavored to present the proposed axis as a conceptual framework rather than an established consensus. Much of the evidence is correlative, and the causal relationships between CRF signaling and the aging hallmarks require further investigation. Alternative explanations for the observed associations, such as glucocorticoid elevation, sympathetic overactivation, sleep disruption, and physical inactivity, have not been systematically dissected from the direct effects of CRF, and these factors likely interact in complex ways to modulate the aging process.

Several unresolved questions limit the translational potential of targeting the CRF-aging axis. First, the dual role of glucocorticoids, which are anti-inflammatory under acute conditions but pro-inflammatory under chronic stress, remains incompletely understood, particularly in the context of the aging brain. Similarly, the opposing actions of CRFR1 and CRFR2 in neuroinflammation present a significant challenge for therapeutic development, as global receptor blockade may yield unpredictable outcomes depending on the tissue context and disease stage. The repeated failures of CRFR1 antagonists in clinical trials for psychiatric disorders, despite promising preclinical data, underscore the translational gap between animal models and human efficacy and should inform more cautious mechanistic interpretations.

The translational limitations from animal models to humans deserve explicit attention. Rodent models, while invaluable for mechanistic studies, may not fully recapitulate the complexity of human brain aging and stress responses. Sex differences in CRF signaling and aging have been documented but are often overlooked; women show greater vulnerability to stress-related disorders and may exhibit differential CRFR1 signaling (Bangasser et al., 2017). Future research must explicitly incorporate sex as a biological variable.

Furthermore, this review has not addressed the emerging role of the microbiome-gut-brain axis as a critical modifier of stress-related aging. The gut microbiota can influence HPA axis activity, systemic inflammation, and even central CRF expression (Sudo et al., 2004). Disruptions in the gut microbiome (dysbiosis) are associated with both aging and stress. Integrating the gut-brain axis into the CRF-aging framework represents a promising and necessary direction for future research.

### 6.2 Future directions

Subsequent investigations should prioritize the following directions. Longitudinal cohort studies are needed to establish a clinical validation system for the CRF-aging axis, systematically assessing peripheral blood CRF levels, CRFR1 expression, and downstream signaling molecules across different age groups to validate this framework in humans. The molecular basis of tissue-specific senescence also warrants attention; single-cell and spatial transcriptomics could clarify how local CRFR1/CRFR2 expression ratios, GR splice variants, and epigenetic backgrounds influence tissue-specific responses. For therapeutic translation, biomarker-guided patient stratification—informed by the rs110402 polymorphism and the clinical success of crinecerfont—should be incorporated into future trials to evaluate efficacy in high-aging-load subgroups, alongside multi-target combinations such as CRFR1 antagonists with senolytics or mitochondrial antioxidants with NAD^+^ precursors. Finally, the reversibility of the CRF-aging axis requires investigation: can intervention in CRF signaling reverse established senescence phenotypes, and if so, what is the optimal window and duration?

## 7 Conclusion

In conclusion, this review identifies CRF as a central molecular hub linking psychological stress to biological aging. It systematically constructs an integrated regulatory network comprising “CRF-mitochondria-inflammation-senescence,” thereby providing a cohesive conceptual framework for understanding the mechanisms through which stress may accelerate aging. The CRF signaling pathway, along with its downstream effector molecules, holds promise as a new generation of candidates for diagnostic markers and therapeutic targets related to aging and associated diseases. The 30-year clinical translation journey from antalarmin to crinecerfont illustrates that, despite numerous challenges in targeted intervention of the CRF system, progress is achievable. With the advent of precision medicine strategies and the investigation of multi-target combination therapies, intervention strategies aimed at the CRF-aging axis may successfully transition from the laboratory to clinical practice in the foreseeable future.
